# Optimal response to tislelizumab plus chemotherapy in metastatic triple-negative breast cancer: a case report and literature review

**DOI:** 10.3389/fonc.2024.1328844

**Published:** 2024-03-28

**Authors:** Yuxin Ma, Xinhong Shi, Kun Zhao, Shuyi Hu, Yue Shi, Yingying Jiang, Yiling Liu, Lin Lu, Yuting Chang, Fei Zhou, Yingying Dai, Zipeng Wu, Shiyi Li, Zhiying Qian, Xia Xu, Chenchen Li, Bo Shen, Guoren Zhou, Cheng Chen, Xiaohua Wang, Jifeng Feng

**Affiliations:** ^1^ Department of Medical Oncology, The Affiliated Cancer Hospital of Nanjing Medical University & Jiangsu Cancer Hospital & Jiangsu Institute of Cancer Research, Nanjing, Jiangsu, China; ^2^ Huaian Hospital of Huaian City, Huaian Cancer Hospital, Huaian, Jiangsu, China; ^3^ Department of Oncology, Province Geriatric Hospital, Nanjing, Jiangsu, China

**Keywords:** metastatic triple negative breast cancer (mTNBC), immune checkpoint inhibitors (ICIS), tislelizumab, eribulin mesylate, case report

## Abstract

Metastatic triple-negative breast cancer (mTNBC) has the worst prognosis among breast cancer subtypes. Immune checkpoint inhibitors (ICIs) plus chemotherapy have promising survival benefits. Herein, we report a 51-year-old woman whose metastatic lesions were diagnosed as triple-negative subtype and who received tislelizumab plus eribulin treatment and achieved excellent efficacy. To our knowledge, this study is the first attempt to present tislelizumab in combination with eribulin for mTNBC treatment. New treatments resulting in prolonged survival and durable clinical responses would benefit mTNBC patients. Then, we summarize the possible influencing factors of the interaction between tislelizumab and eribulin.

## Introduction

Breast cancer (BC) is the most prevalent tumor and the main cause of women’s deaths worldwide (1). Triple-negative breast cancer (TNBC), accounting for 15%–20% of all BC types, is defined as the absence of estrogen receptor (ER), progestogen receptor (PR), and human epidermal growth factor receptor 2 (HER2) (2) and has the worst prognosis (3). Because TNBC is insensitive to endocrine therapy and anti-HER2 therapy, till now, chemotherapy is still the standard treatment as the guidelines highly recommend.

In the past 10 years, immune checkpoint inhibitors (ICIs) achieved huge advances in cancer immunotherapy. Tislelizumab as a PD-1 inhibitor has a large overlap between the binding surface of PD-1 and PD-L1 and an extremely low dissociation rate compared to PD-1 (4, 5). This indicates that tislelizumab has higher targeting affinity and efficacy.

Eribulin mesylate (eribulin) is a microtubule dynamics inhibitor. Preclinical studies showed that eribulin could promote mitosis, reverse epithelial-to-mesenchymal transition, and induce vascular remodeling (6–10). Compared with other chemotherapy, eribulin significantly prolonged the median overall survival (OS) in the metastatic TNBC (mTNBC) subgroup (11, 12).

This case report presents an excellent response from a patient with mTNBC who received tislelizumab plus eribulin after the failure of first-line treatment.

## Case presentation

**Figure 1:**
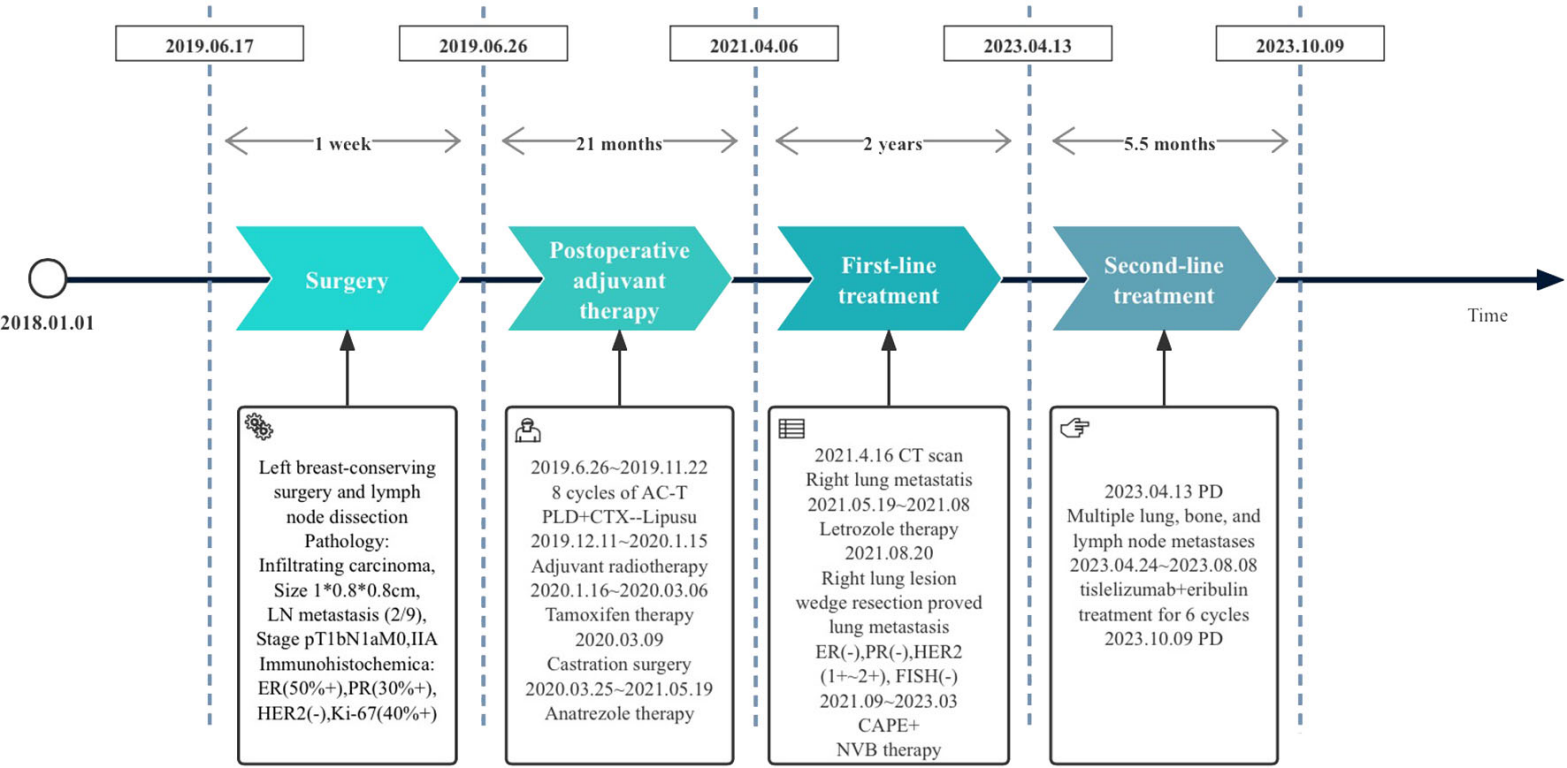
Treatment flowchart: PLD, pegylated liposomal doxorubicin; CTX, cyclophosphamide; Lipusu, paclitaxel liposome; CAPE, capecitabine; NVB, vinorelbine; PD, disease progression.

**Figure 2:**
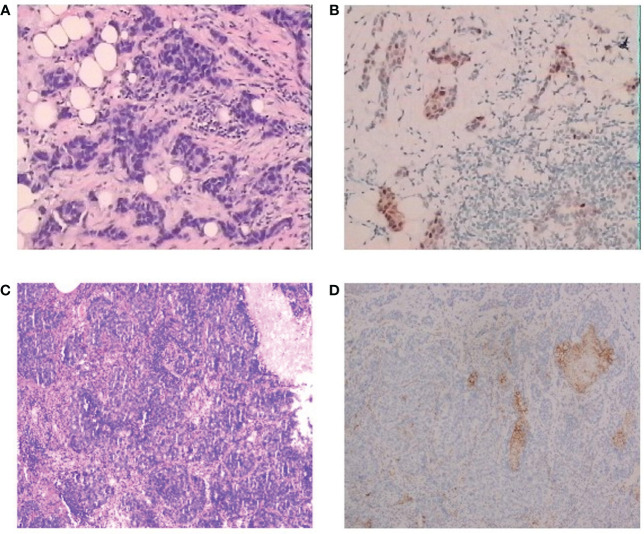
Pathological findings for the patient. **(A)** Hematoxylin and eosin staining of the tumor, diagnosed as invasive ductal carcinoma, H&E, ×100. **(B)** Immunohistochemistry of primary tumor, H&E, ×100. **(C)** Immunohistochemistry of lung metastasis, H&E, ×100. **(D)** Immunohistochemistry of PD-L1 of the lung metastasis, H&E, ×100.

A 51-year-old female patient presented to our cancer center with the complaint of a mass at the left breast in the upper outer quadrant since October 2018 (**Figure 1**). On June 17, 2019, this patient received left breast-conserving surgery and lymph node dissection in our hospital. Postoperative pathology test showed non-specific Grade II infiltrating ductal carcinoma, tumor size of 1 * 0.8 * 0.8 cm, and axillary lymph node metastasis (2/9). Immunohistochemical examination results showed ER (50%+), PR (30%+), HER2 (−), and Ki-67 (40%+) (**Figures 2A, B**). The patient was diagnosed with left HR+HER2− BC (pT1bN1aM0, stage IIA). Then, the patient received eight cycles of AC-T treatment, local radiotherapy, and endocrine therapy.

**Figure 3:**
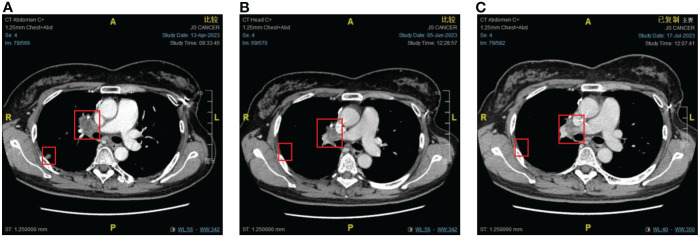
The response of lung metastases to immunotherapy combined with chemotherapy. **(A)** Before combination therapy. **(B)** After two cycles of treatment. **(C)** After four cycles of treatment.

On May 19, 2021, a right lung nodule was discovered; thus, endocrine therapy was changed to letrozole. Wedge resection of right lung lesions was performed on August 20, 2021. Postoperative pathology revealed metastatic lung lesions from breast cancer. Immunohistochemical examination results showed ER (−), PR (−), HER2 (1+~2+), and Ki-67 (60%+). Fluorescence *in situ* hybridization (FISH) detection showed no HER2 amplification. PD-L1 combined positive score (CPS) was 3 points (**Figures 2C, D**). The clinical diagnosis was mTNBC. From September 2021 to January 2022, the patient was given “capecitabine plus vinorelbine” treatment and then received capecitabine or vinorelbine maintenance therapy (**Figure 3**).

**Figure 4:**
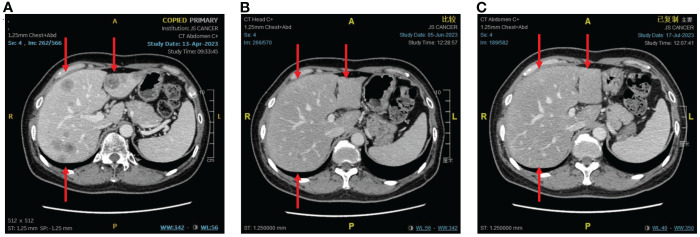
The response of liver metastases to immunotherapy combined with chemotherapy. **(A)** Before combination therapy. **(B)** After two cycles of treatment. **(C)** After four cycles of treatment.

An imaging examination on April 13, 2023, indicated progressive disease (PD) with multiple metastases all over her lungs, liver, bones, and lymph nodes. From April 24, 2023, to August 8, 2023, the patient received six cycles of “tislelizumab (200 mg d0/q3w) plus eribulin (2 mg d1,8/q3w)”. The latest CT scan was on July 17, 2023, and indicated that after four cycles of treatment, only one mass was left in the right lung (1.68 * 0.96 cm). The relief rate was more than 95% (**Figures 3**, **4**). However, after six cycles of treatment, the patient developed new brain metastases. The progression-free survival (PFS) of this treatment was 5.5 months, and the lung lesions were almost absent.

**Figure 5:**
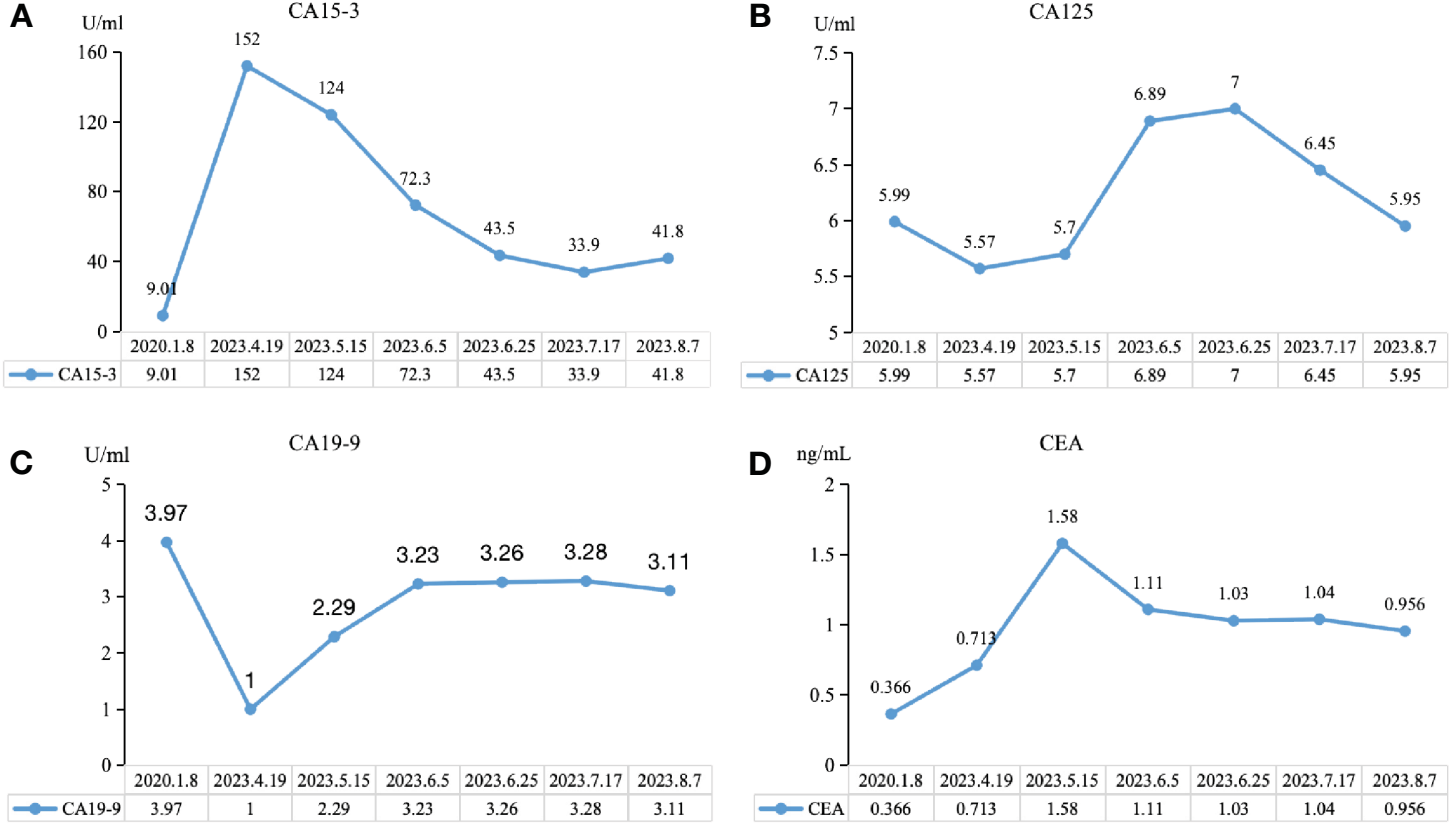
Trend chart of changes in tumor biomarkers before and after immunotherapy. **(A)** CA15-3, carbohydrate antigen 15-3. **(B)** CA19-9, carbohydrate antigen 19-9. **(C)** CA125, carbohydrate antigen 125. **(D)** CEA, carcinoembryonic antigen.

We observed the changes in tumor biomarkers and found that with the progress of immunotherapy, the level of carbohydrate antigen 15-3 (CA15-3) significantly decreased, while the level of carbohydrate antigen 19-9 (CA19-9) achieved a certain extent increase and remained at a lower normal level. Both carbohydrate antigen 125 (CA125) and carcinoembryonic antigen (CEA) increased at first and then decreased (**Figure 5**). Tumor biomarkers were greatly affected by changes in the individual’s internal environment. Therefore, more clinical studies are needed to confirm whether the trend of changes in this case is a universal phenomenon and whether it has specific guiding significance for the selection of treatment plans and efficacy.

## Discussion

In this case, after failures of chemotherapy and endocrine therapy, the patient showed an excellent response to “tislelizumab plus eribulin” treatment, suggesting that tislelizumab could be used effectively to treat mTNBC.

TNBC is insensitive to endocrine therapy and targeted therapy, and the typical treatment mode is still chemotherapy. The 5-year survival rate of patients with stage III–IV TNBC is 13%, significantly lower than that of other subtypes (13). After receiving chemotherapy, anti-HER2-targeted therapy, or endocrine comprehensive treatment, the median survival time of patients with advanced non-TNBC can be as long as 48–60 months, while the median survival time of patients with mTNBC is only 10–13 months (4, 14).

Previous studies indicated that TNBC has the highest expression of PD-L1 (approximately 40%) in both tumor cells and immune cells (2, 15–18), which can provide more targets for ICI and enhance the efficacy of immunotherapy (10, 19, 20). In the era of immune monotherapy clinical trials (21–23), only a fraction of patients could benefit from PD-1/PD-L1 inhibitor monotherapy (24). This may be due to the activities of ICIs relying on the interaction with T cells. PD-L1 binding to PD-1 may facilitate tumor immunity by suppressing cytotoxic T-cell responses (14). Higher levels of baseline T-cell infiltration are more likely to respond to ICI treatment (25, 26). In order to improve the clinical efficacy, tislelizumab is specifically engineered to minimize FcR binding on macrophages, thereby abrogating antibody-dependent cell-mediated phagocytosis (ADCP), ultimately avoiding T-cell depletion and enhancing its antitumor activity (5, 27, 28).

Tislelizumab, as a humanized immunoglobulin G4 (IgG4) PD-1 inhibitor, has a large overlap between the binding surface of PD-1 and PD-L1 and an extremely low dissociation rate compared with PD-1 (4, 5). PD-1 is one of the co-inhibitory immune checkpoint (ICP) receptors induced upon T-cell activation and widely expressed (70.3%) in tumor-infiltrating lymphocytes (TILs) (29). Of TNBC tumors, 21.9% comprise more than 50% of TILs (30). Studies of TNBC have shown a positive correlation between PD-1 expression in TILs and better clinical outcomes (31–33). The escape of tumor cells from T-cell killing was mediated by PD-1 by transducing negative signaling of effector T-cell activity by the interaction with PD-L1 (34, 35).

PD-1/PD-L1 inhibitors and chemotherapy may have synergistic effects *in vivo* (7, 11). Patients who received immunotherapy plus chemotherapy achieved higher OS and controllable adverse event risks (12, 20, 36). The combination of ICIs with chemotherapy has become a research hotspot to improve the clinical efficacy of ICIs.

The IMpassion130 trial proved that patients can benefit from atezolizumab combined with nab-paclitaxel, especially patients with PD-L1 expression (36). However, in IMpassion131, the immunotherapy subgroup obtained worse OS than the placebo subgroup (20). The conflicting results directly lead Roche to withdraw the indication of atezolizumab in mTNBC. Pembrolizumab plus chemotherapy significantly improved the PFS of mTNBC patients with PD-L1 positive score (CPS) ≥10 in the KEYNOTE-355 trial. Therefore, the 2023 Chinese Society of Clinical Oncology (CSCO) guidelines recommend PD-1 inhibitors combined with chemotherapy for mTNBC rescue treatment. In the ENHANCE1 trial, eribulin along with pembrolizumab was given to patients who had mTNBC and ≤2 prior systemic anticancer therapies in the metastatic setting. The results showed an objective response rate (ORR) of 23.4% (95% CI: 17.2–30.5), and the median OS of mTNBC patients who received “pembrolizumab or placebo plus eribulin” as first- to third-line therapy was 16.1 versus 11.1 months (3, 37). The ENHANCE1 study provides strong evidence supporting the application of eribulin combined with PD-1 inhibitors in mTNBC patients and the further clinical development of eribulin plus pembrolizumab as a potential antitumor strategy for mTNBC patients (**Table 1**).

**Table 1:** Clinical trials of PD-1/PD-L1 checkpoint inhibitor therapy in metastatic triple-negative breast cancer.

Trial	Patient	Regimen	N	PD-1/PD-L1 group	Placebo group
IMpassion130 randomized, double-blind, phase III (NCT02425891)	Untreated, unresectable, locally advanced, or metastatic TNBC	nP 100 mg/m^2^ +/− A 840 mg q28d	451 vs. 451	ITT mOS: 21 m mPFS: 7.2 m PD-L1+ mOS: 25.4 m mPFS: 7.5 m AE: 58.7%	ITT mOS: 18.7 m mPFS: 5.5 m PD-L1+ mOS: 17.9 m mPFS: 5.0 m AE: 41.6%
IMpassion131 randomized, double-blind, phase III (NCT03125902)	Untreated, unresectable, locally advanced, or metastatic TNBC	A 840 mg +/− P 100 mg/m^2^ q28d	434 vs. 217	ITT mOS: 19.2 m mPFS: 5.7 m PD-L1+ mOS: 22.1 m mPFS: 6 m	ITT mOS: 22.8 m mPFS: 5.6 m PD-L1+ mOS: 28.3 m mPFS: 5.7 m
KEYNOTE-355 randomized, double-blind, phase III (NCT02819518)	Untreated, unresectable, locally advanced, or metastatic TNBC	nP 100 mg/m^2^ /P 90 mg/m^2^ /Gem 1,000 mg/m^2^ +cp q28d +/− Pem 200 mg q3w	566 vs. 281	CPS ≥ 10 mOS: 23.0 m mPFS: 9.7 m CPS ≥ 1 mOS: 17.6 m mPFS: 7.6 m ITT mOS: 17.2 m mPFS: 7.5 m	CPS ≥ 10 mOS: 16.1 m mPFS: 5.6 m CPS ≥ 1 mOS: 16.0 m mPFS: 5.6 m ITT mOS: 15.5 m mPFS: 5.6 m
ENHANCE-1 phase Ib/II (NCT02513472)	Metastatic TNBC 1–2 prior systemic anticancer therapies	Pem 200 mg + Eribulin 1.4 mg/m^2^ q3w	167	Stratum 1 PD-L1+ mOS: 21.0 m mPFS: 6.1 m mDOR: 8.3 m ORR: 34.5% PD-L1− mOS: 15.2 m mPFS: 3.5 m mDOR: 15.2 m ORR: 16.1%	Stratum 2 PD-L1+ mOS: 14.0 m mPFS: 4.1 m mDOR: 8.2 m ORR: 24.4% PD-L1− mOS: 15.5 m mPFS: 3.9 m mDOR: 8.6 m ORR: 18.2%

TNBC, triple-negative breast; A, atezolizumab; nP, Nab-paclitaxel; Pem, pembrolizumab; Gem, gemcitabine; cp, carboplatin; mOS, median overall survival; mPFS, median progression-free survival; AE, adverse events; mDOR, median duration of response; ORR, objective response rate; ITT, intention to treat; CPS, PD-L1 expression combined positive score; PD-1, programmed death 1; PD-L1, programmed death-ligand 1.

**Figure 6:**
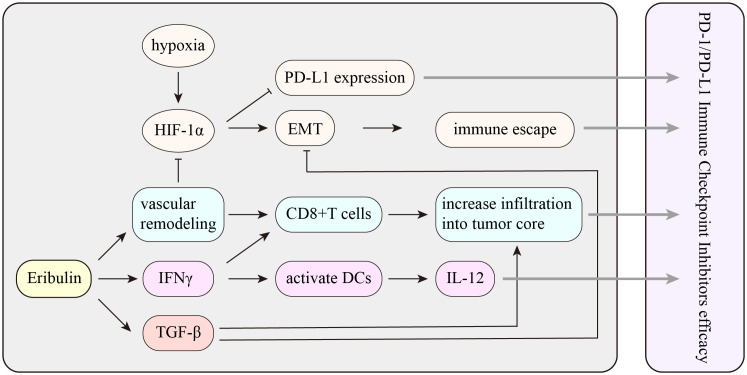
The potential mechanism of the interaction between eribulin and PD-1/PD-L1 immune checkpoint inhibitors. HIF-1α, hypoxia-inducible factor 1α; EMT, epithelial-to-mesenchymal transition; TGF-β, transforming growth factor­β; IFNγ, interferon γ.

The synergistic effect between tislelizumab and eribulin is currently only at the theoretical level, and more experiments are needed. Previous reports proved that eribulin in combination with anti-PD-1 antibody showed marked antitumor activity, while 4T1#31 tumors failed to respond to anti-PD-1 antibody. Therefore, eribulin may inhibit an immune evasion mechanism involving immune checkpoints. Eribulin has been observed to have immunomodulatory activity *in vivo*, and the function of inducing vascular remodeling may be the unique activity of eribulin. It was reported that eribulin induced reoxygenation by vascular remodeling to improve the hypoxic condition in mTNBC patients (10, 38). In addition, immunosuppressive factor transforming growth factor­β (TGF-β) was evaluated in terms of pSmad2 expression, which is typically associated with hypoxic conditions, and showed a decrease after eribulin treatment. TGF-β is crucial to epithelial-to-mesenchymal transition (EMT) and immune regulation, including the induction of regulatory T cells and the suppression of effector T cells. Eribulin can enhance the antitumor immune response by inhibiting the EMT and improving the immune microenvironment in breast cancer. Several retrospective studies proposed that eribulin promoted an antitumor immune response by increasing CD8+ T-cell and NK cell infiltration and inhibiting EMT (38, 39). However, CD8+ T cells prevent tumor progression by causing extracellular vesicle (EV)-mediated depletion of mesenchymal tumor within the tumor environment and stromal cells (40). The number of TILs has been reported as a predictive marker for eribulin responders (41). Eribulin activates interferon (IFN) signaling, and the activation of IFNγ signaling likely plays a key role in the increased infiltration of activated T cells and NK cells into tumors. Moreover, IFNγ also contributes to the efficacy of ICIs. Compared with those of non-responders, patients who respond to ICIs usually have higher expression scores of IFNγ-related genes (42). Therefore, activation of IFNγ signaling by eribulin could improve patients’ response to ICIs (39) (**Figure 6**).

Although tislelizumab has been observed to have good efficacy in several solid tumors (15, 43–45), its therapeutic efficacy in mTNBC has not been reported before. The patient reported in this case achieved great response to “tislelizumab plus eribulin” treatment, and no high grade of adverse event was observed, confirming the therapeutic potential of this regimen in mTNBC. Given that this is only a case report and the lack of long-term follow-up data, the clinical efficacy of this treatment regimen remains to be further confirmed by more clinical practice.

## Conclusion

This case report is the first to describe that one mTNBC patient in our cancer center treated with “tislelizumab plus eribulin” after multi-line therapy achieved significant pathological remission. Tislelizumab plus eribulin seems to be a novel promising regimen in TNBC. However, eribulin may have a potential activity to enhance the efficacy of ICIs, and further studies are crucial to clarify the interactions between tislelizumab and eribulin in mTNBC.

## Data availability statement

The original contributions presented in the study are included in the article/supplementary materials. Further inquiries can be directed to the corresponding authors.

## Ethics statement

The studies involving humans were approved by Ethics Committee of Jiangsu Cancer Hospital Affiliated with Jiangsu Cancer Hospital. The studies were conducted in accordance with the local legislation and institutional requirements. The participants provided their written informed consent to participate in this study. Written informed consent was obtained from the individual(s) for the publication of any potentially identifiable images or data included in this article.

## Author contributions

YM: Conceptualization, Data curation, Formal analysis, Investigation, Methodology, Writing – original draft. XS: Conceptualization, Data curation, Investigation, Project administration, Writing – original draft. KZ: Data curation, Investigation, Project administration, Writing – original draft. SH: Project administration, Resources, Writing – original draft. YS: Data curation, Investigation, Writing – original draft. YJ: Data curation, Methodology, Writing – original draft. YL: Data curation, Investigation, Writing – original draft. LL: Data curation, Investigation, Writing – original draft. YC: Data curation, Investigation, Writing – original draft. FZ: Data curation, Investigation, Writing – original draft. YD: Data curation, Methodology, Writing – original draft. ZW: Data curation, Methodology, Writing – original draft. SL: Data curation, Methodology, Writing – original draft. ZQ: Conceptualization, Writing – review & editing. XX: Conceptualization, Writing – original draft. CL: Conceptualization, Writing – review & editing. BS: Conceptualization, Writing – review & editing. GZ: Conceptualization, Writing – review & editing. CC: Conceptualization, Funding acquisition, Project administration, Resources, Supervision, Validation, Writing – review & editing. XW: Conceptualization, Funding acquisition, Project administration, Resources, Supervision, Validation, Writing – review & editing. JF: Conceptualization, Funding acquisition, Resources, Supervision, Validation, Writing – review & editing.
